# The glutamine antagonist prodrug JHU-083 slows malignant glioma growth and disrupts mTOR signaling

**DOI:** 10.1093/noajnl/vdaa149

**Published:** 2020-10-29

**Authors:** Alex Shimura Yamashita, Marina da Costa Rosa, Vittorio Stumpo, Rana Rais, Barbara S Slusher, Gregory J Riggins

**Affiliations:** 1 Department of Neurosurgery, Johns Hopkins University School of Medicine, Baltimore, Maryland, USA; 2 Johns Hopkins Drug Discovery, Department of Neurology, Johns Hopkins University School of Medicine, Baltimore, Maryland, USA

**Keywords:** cell cycle, glioma, glutamine metabolism, IDH mutation, mTOR signaling

## Abstract

**Background:**

Metabolic reprogramming is a common feature in cancer, and it is critical to facilitate cancer cell growth. *Isocitrate Dehydrogenase 1/2 (IDH1* and *IDH2)* mutations (*IDH*mut) are the most common genetic alteration in glioma grade II and III and secondary glioblastoma and these mutations increase reliance on glutamine metabolism, suggesting a potential vulnerability. In this study, we tested the hypothesis that the brain penetrant glutamine antagonist prodrug JHU-083 reduces glioma cell growth.

**Material and Methods:**

We performed cell growth, cell cycle, and protein expression in glutamine deprived or *Glutaminase* (*GLS*) gene silenced glioma cells. We tested the effect of JHU-083 on cell proliferation, metabolism, and mTOR signaling in cancer cell lines. An orthotopic *IDH1R132H* glioma model was used to test the efficacy of JHU-083 in vivo.

**Results:**

Glutamine deprivation and *GLS* gene silencing reduced glioma cell proliferation in vitro in glioma cells. JHU-083 reduced glioma cell growth in vitro, modulated cell metabolism, and disrupted mTOR signaling and downregulated Cyclin D1 protein expression, through a mechanism independent of TSC2 modulation and glutaminolysis. IDH1R132H isogenic cells preferentially reduced cell growth and mTOR signaling downregulation. In addition, guanine supplementation partially rescued *IDH*mut glioma cell growth, mTOR signaling, and Cyclin D1 protein expression in vitro. Finally, JHU-083 extended survival in an intracranial *IDH1* mut glioma model and reduced intracranial pS6 protein expression.

**Conclusion:**

Targeting glutamine metabolism with JHU-083 showed efficacy in preclinical models of *IDH*mut glioma and measurably decreased mTOR signaling.

**Key Points**

- The glutamine antagonist prodrug, JHU-083, inhibits glioma cell growth.
- Antagonizing glutamine metabolism with JHU-083 inhibits mTOR signaling.
- JHU-083 extends survival in an orthotopic glioma model.

**Importance of the Study**

Gliomas are the most frequent primary brain malignancy and although there has been steady progress in improved therapy, most patients with a diagnosis of malignant glioma die of the disease. A brain penetrant glutamine antagonist prodrug, JHU-083, extends survival in an intracranial model of IDH mutant glioma. The mechanism of action of JHU-083 is associated with mTOR signaling downregulation and impairment of purine biosynthesis. This study provides support for targeting glutamine metabolism as an effective way to slow growth or progression of *IDH* mutant malignant gliomas, a potentially lethal brain tumor of young adults.

Gliomas, the most frequent brain cancer, are a group of genetically diverse nervous system tumors arising from glial cells. With decades of research and progress in surgery, radio and chemotherapy survival for malignant glioma patients has steadily increased, but only incrementally. Seminal studies showed that the mutually exclusive *Isocitrate Dehydrogenase 1/2* (*IDH1* and *IDH2*) mutations are the most common genetic alteration in glioma (grade II and III) and *IDH*mut glioblastoma (grade IV) that normally arises via progression from the lower grades.^1^ Subsequent studies have identified *IDH1/2* mutation in several cancer types such as leukemia, chondrosarcomas, melanoma.^2^ Recently, World Health Organization had advanced the glioma classification according to the presence of *IDH*mut and grade, in addition to histological features.^3^  *IDH1/2* mutation in the catalytic pocket generates a neomorphic enzyme, which converts αKG and NADPH to (*R*)-2hydroxyglutarate (2-HG).^2^ The *IDHmut* and the accumulation of the “oncometabolite” 2-HG are associated with hypermethylation phenotype, called glioma CpG island methylator phenotype, through competitive inhibition of Ten-eleven Translocation enzyme and JmjC family of histones deacetylases.^2,4^

Metabolism is altered to promote malignant growth and survival in many cancers and has been considered as a potential therapeutic target. A body of evidence demonstrates that not only glucose, but also glutamine is an essential metabolic pathway for cancer cell growth.^5^ Glutamine plays an important role in nucleotide, lipid, and protein synthesis, redox balance, and supports increased demand for ATP. Glutamine metabolism importance is underscored by the robust efficacy afforded by (glutaminase) GLS inhibitors/glutamine antagonist in preclinical cancer studies^6–9^ and a recent phase I clinical trial for *IDH* mut glioma testing GLS inhibitor (NCT03528642). Glutamine metabolism is controlled by several oncogenes and tumor suppressors. In glioblastoma, activation of PI3K/AKT/mechanistic target of rapamycin (mTOR) signaling by EGFRvIII mutation induces high dependence of glutamine metabolism and treatment with mTOR and GLS inhibitors induced synergistic cell death.^7^ In addition, glioblastoma cells have high dependence of glutamine for nucleotide synthesis and lower dependence for TCA cycle anaplerosis in glutamine-deprived conditions, suggesting that GLS inhibition may not be enough to reduce glioblastoma growth.^10^ In addition, *IDH1* mutant glioma cells are highly dependent on glutamine metabolism and are more responsive to GLS inhibitors/glutamine antagonists or *GLS* gene silencing.^11,12^

Considerable effort has been directed to the development of metabolic inhibitors for cancer therapy. 6-diazo-5-oxy-L-norleucin (DON) is an irreversible glutamine inhibitor that showed initial clinical benefit, but further trials showed limited clinical application due to its toxicity.^6^ Although glutamine metabolism has been shown as a potential target therapy in several cancer types, few studies have addressed the impact of glutamine antagonist in *IDH1R132H* mutant gliomas in vivo. Taking the advantage of DON’s antiproliferative properties, in this study, we explore the effect of a brain penetrant DON prodrug, named JHU-083, in a preclinical model of *IDH1*mut mouse model. *IDH*mut glioma reliance on glutamine metabolism has led us to hypothesize that targeting glutamine metabolism via JHU-083 could reduce cell proliferation and extend survival in vivo for mice bearing these intracranial malignancies.

## Materials and Methods

### Cell Culture and Reagents

Cell culture was performed as previously described.^13^ Detailed cell culture conditions, gene silencing, and reagents are described in the Supplementary Material and Methods. Glutamine antagonist JHU-083 (Ethyl 2-(2-Amino-4-methylpentanamido)-DON) and JAM326 (Isopropyl 2-(2-Amino-4-methylpentanamido)-DON) synthesis were conducted by Redicius s.r.o (Czech Republic) as previously described.^14^ For in vitro experiments, JHU-083 and JAM326 were diluted in HEPES buffered saline.

### Cell Viability, Colony Formation, Neurosphere Formation Assay, and 5-Bromo-2′-Deoxyuridine Incorporation Assay, and Metabolites Quantification

Cell viability was assessed using the fluorescent alamarBlue cell proliferation assay (ThermoFisher Scientific) as instructed by the manufacturer. Briefly, single suspension cell or adherent viable cells were plated in 96-well black flat-bottom plates and treated with the indicated drug concentration. Fluorescence was read in Victor-3 automated plate reader (Perkin-Elmer), with a 540-nm excitation/590-nm emission filter. For colony formation assays, cells were treated 24 h after plating and media was changed every 3–4 days. Cells were incubated for 10–12 days until visible colonies were observed. Cells were washed in phosphate-burred saline (PBS), fixed in 4% paraformaldehyde, and stained with crystal violet 0.5%. Colonies were counted under a dissecting microscope. For neurosphere formation assay, neurospheres were dissociated in single cells, plated in 96-well plates in serum-free media with indicated drug, and neurospheres were counted 12–14 days after drug exposure. Luminescent 5-bromo-2′-deoxyuridine (BrdU) incorporation assay was conducted according to the manufacturer’s instruction (Roche). Florescence and luminescence was read in Victor-3 automated plate reader (Perkin-Elmer). Metabolite quantification is described in the Supplementary Material and Methods.

### Western Blot and Immunohistochemistry

Detailed Western blot and immunohistochemistry (IHC) procedures and antibodies are described in the Supplementary Material and Methods.

### Animal Studies

All animal protocols and procedures were performed in accordance with the Johns Hopkins Animal Care and Use Committee Guidelines. Female athymic nude mice (6–8 weeks), purchased from National Cancer Institute, were kept in a pathogen-controlled environment with access to food and water ad libitum. After mice adaptation (2 weeks) at animal facility, orthotopic glioma xenografts were performed as previously described.^15^ BT142 cells (3 × 10^5^) were orthotopically implanted in nude mice fixed in a stereotaxic frame. Mice were randomized and treatment was initiated 5 days after tumor implantation. JHU-083 was diluted in PBS immediately before treatment and administered through intraperitoneal injection until the end of experiment at the indicated dose and regimen. Low-dose JHU-083 (1.9 mg/kg) was administered 5 days/week for 3 weeks and then 2 days/week. High-dose JHU-083 (25 mg/kg) was administered 2 days/week. Animals were monitored daily and euthanized in accordance with the Johns Hopkins Animal Care and Use Guidelines.

### Statistical Analysis

The statistical analysis was performed using GraphPad Prism 8.4 (GraphPad Software Inc.). The data were expressed as mean ± SEM. Student’s *t*-test was used to compare two means. One-way or 2-way analysis of variance followed by Tukey’s Multiple Comparison and Bonferroni post-tests were used for multiple comparisons when appropriated, respectively. Log-rank (Mantel-Cox) test was used to analyze Kaplan–Meier survival curves. *P* < .05 was considered as statistically significant.

## Results

### Glutamine Is Required for Glioma Cell Growth

**Figure 1.**
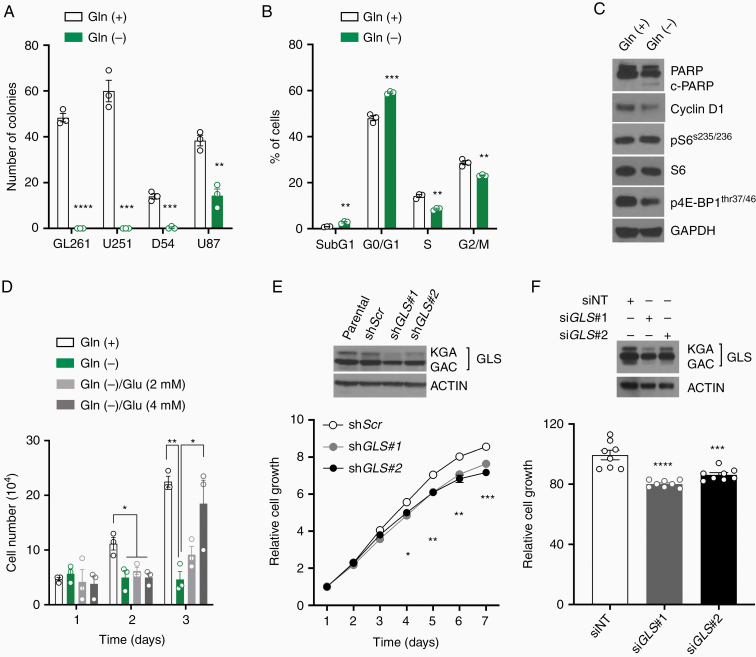
Glioma cells require glutamine for cell proliferation. (A) Colony-forming assays were performed in glioma cells cultivated in glutamine-free media. (B and C) U251 were cultivated in glutamine-free media (24 h) and cell cycle and protein expression were analyzed. (D) U251 cell growth cultivated in glutamine-free media supplemented with Glu. (E and F) Constitutive and transient shRNA and siRNA-mediated *GLS* silencing, respectively. GLS protein expression was analyzed by Western blot. Cell viability was conducted over 7 days (shRNA) or 3 days after transfection (siRNA). *P < .05; **P < .01; ***P < .001; ****P < .0001 versus Control. Gln: glutamine. Glu: glutamate. siNT: small interfering Non-target control. Gln (+): glutamine (4 mM). Gln (−): glutamine-free media. Glu: glutamate.

Complete glutamine deprivation strongly reduced colony numbers in glioma cells. U87 cell line was the most resistant cell line, reducing only by 54% in colonies number after complete glutamine deprivation (Figure 1A). Glutamine deprivation reduced glioblastoma cells proliferation in a dose-dependent fashion (Supplementary Figure 1A). The effect of glutamine deprivation was further characterized in a U251 cell line. Cell cycle analysis showed G0/G1 cell cycle arrest and reduction in cells in S and G2/M cell cycle phase with glutamine deprivation (Figure 1B). Cleaved-PARP was slightly increased and Cyclin D1 and phosphorylated 4E-BP1(thr37/46) protein expression were reduced in the glutamine deprivation (Figure 1C). Glutamate (Glu) supplementation in glutamine-deprived media restored U251 cell growth (Figure 1D). U251 viable cells and colony formation were partially restored with Glu supplementation in glutamine-deprived media (Supplementary Figure 1B and C). To understand specifically the impact of glutaminolysis in cell proliferation, we generated stable and transient *GLS* gene silencing, with 2 distinct shRNA or siRNA, respectively. Both stable and transient *GLS* gene silencing isoforms (*KGA* and glutaminase C [*GAC*]) slightly reduced U251 cells, compared with respective transfection controls, likely due the preferential reduction in kidney-type glutaminase (KGA) isoform protein expression (Figure 1E and F).

Heterotrophic cancer cells can reciprocally control glucose and glutamine consumption in vitro and this mechanism explains, at least in part, why some cell lines are resistant to glutamine deprivation. Glutamine deprivation did not modulate glucose analog (2-NBDG) uptake and glucose-free media did not affect cell growth (Supplementary Figure 1D and E). Furthermore, glutamine-free media supplemented with increased glucose concentration or cells treated with 2-deoxy-D-glucose, which inhibits hexokinase activity, did not have an effect on cell proliferation (Supplementary Figure 1F), suggesting that glucose metabolism alteration has limited impact in cell growth in this context.

### Targeting Glutamine Metabolism Reduces Glioma Cell Growth

**Figure 2.**
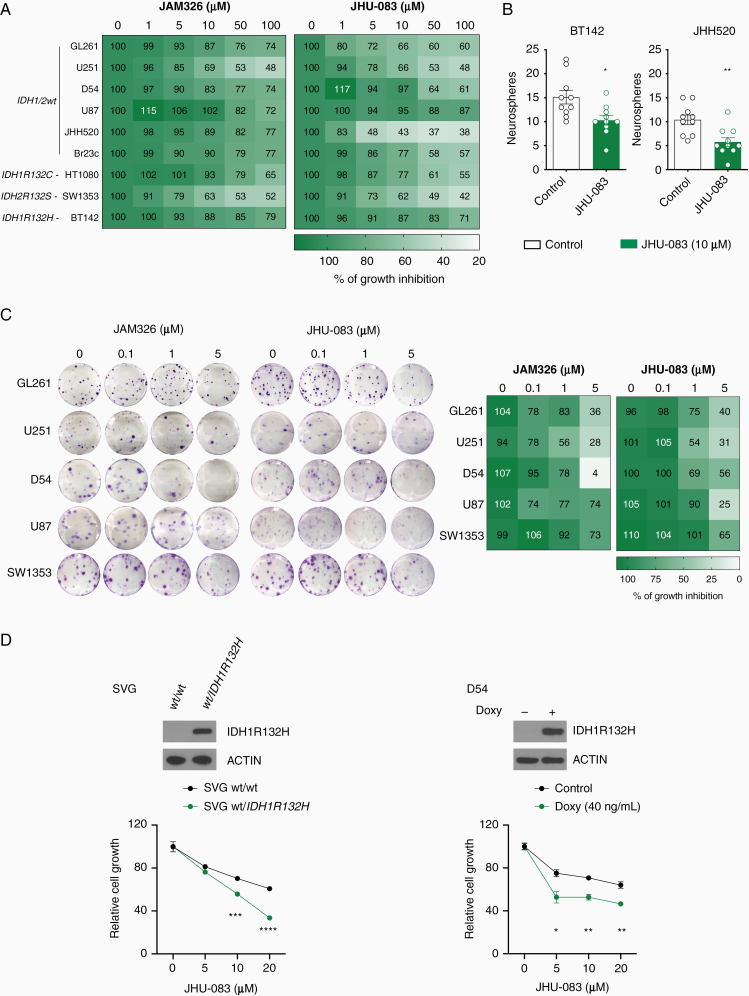
Targeting glutamine metabolism reduces glioma cell growth. (A) Cell viability was performed in cancer cells treated with JAM326 or JHU-083 (72 h). Cell viability is visualized by a color scale. (B) Neurosphere forming assay was performed in cells cultured in serum-free media and treated with JHU-083. (C) Colony-forming assay was performed in adherent glioblastoma cells treated with JAM326 or JHU-083. Colony numbers are visualized by a color scale. (D) Cell viability was performed in IDH1R132H isogenic cells treated with JHU-083 (96 h). IDH1R132H protein expression was analyzed by Western blot. *P < .05; **P < .01; ***P < .001; ****P < .0001 versus Control. Doxy: doxycycline.

Variable GLS (KGA and GAC) protein expression was observed in cancer cell lines (Supplementary Figure 2A). Gene expression analysis detected *GLS1* isoforms (*KGA* and glutaminase C [*GAC*]), using specific primer sets, and *GLS2* mRNA in JHH520, TS603, BT142 glioma neurospheres and in JHH273 xenograft tumor. We also detected stem-like cell markers *NES* and *SOX2* gene expression in these cells (Supplementary Figure 2B). We have previously shown that isogenic glioblastoma cells expressing *IDH1R132H* mutant are more sensitive to GLS pharmacological inhibition or *GLS* gene silencing.^11^ In addition, GLS pharmacological inhibition preferential inhibits *IDH1/2* mutant patient-derived AML cells proliferation in vitro.^16^ Therefore, we explored the direct effect of 2 glutamine antagonist prodrugs (JAM326 and JHU-083) in genetically diverse cancer cells. Both prodrugs showed a dose-dependent antiproliferative effect in all cancer cell lines tested, including *IDH1*wt adherent serum-cultured glioblastoma cells (GL261, U251, D54, and U87), *IDH1*wt suspension serum-free glioblastoma cells (Br23c and JHH520), *IDH1R132H*mut suspension serum-free glioma cells (BT142), HT1080 fibrosarcoma and SW1353 chondrosarcoma cells, harboring *IDH1R132C* and *IDH2R132S* mutations, respectively (*P* < .05 to *P* < .0001; Figure 2A). Reduction in neurosphere formation assay was observed in JHU-083-treated glioma cells (Figure 2B). Antiproliferative dose-dependent effect was observed in all cell lines exposed with both JAM326 and JHU-083 glutamine antagonists in colony formation assay (Figure 2C). Furthermore, we tested the effect of JHU-083 on cell proliferation in 2 isogenic IDH1R132H cell lines. Heterozygous IDH1R132H expression in human normal astrocyte SVG cells^17^ and doxycycline-inducible IDH1R132H expression in D54 glioma cell line^11^ showed reduced cell proliferation when exposure to JHU-083 (Figure 2D).

### JHU-083 Reduces Cell Growth Independently of Glutaminolysis

**Figure 3.**
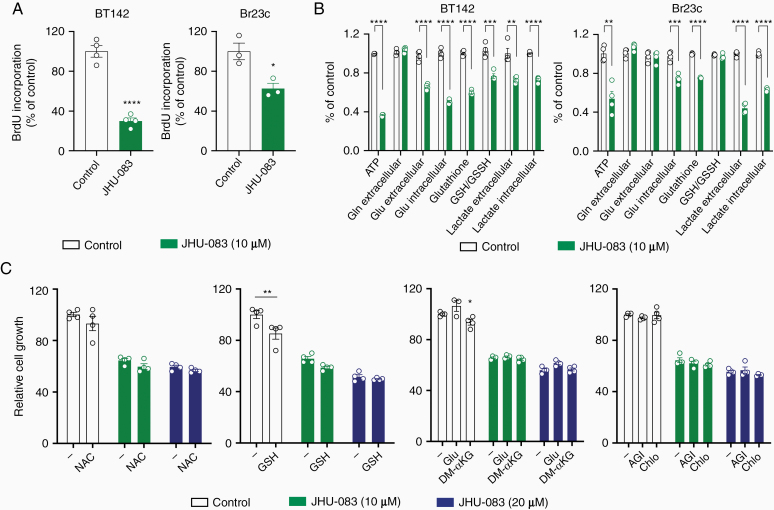
JHU-083 reduces cell growth independently of glutaminolysis. (A) BrdU incorporation into DNA was analyzed in glioma cells treated with JHU-083 (24 h). (B) ATP, Gln, Glu, glutathione, GSH/GSSH ratio, and lactate levels were determined in glioma cells treated with JHU-083 (24 h). (C) Cell viability were performed in BT142 treated with JHU-083 in combination with NAC (4 mM), GSH (4 mM), Glu (4 mM), DM-αKG (10 mM), AGI-5198 (1 µM), and Chloroquine (20 µM) for 72 h. AGI: AGI-5198. Chlo: Chloroquine. *P < .05; **P < .01; ***P < .001; ****P < .0001 versus Control.

JHU-083 has been shown effective in several preclinical cancer and central nervous system disease in vivo models.^8,14,18^ Therefore, we focused in characterizing the effect of JHU-083 in glioma cells. DNA synthesis assessed by BrdU incorporation into DNA was decreased with JHU-083 treatment in BT142 and Br23c cell lines (Figure 3A). JHU-083 treatment reduced ATP, Glu, and lactate in both BT142 and Br23c cells (Figure 3B). Glutathione was reduced in BT142 and Br23c after JHU-083 treatment, but GSH/GSSH ratio was downregulated only in BT142 cells. These data showed that JHU-083 deregulates cell metabolism in glioma cells. Glutaminolysis is essential to provide precursors for glutathione synthesis and to sustain anaplerosis in tricarboxylic citric acid (TCA) cycle in cancer cells. Therefore, we tested if replenishing the antioxidative system or glutaminolysis/TCA intermediates rescued cell proliferation in the presence of JHU-083. Antioxidant compounds (NAC and GSH) or glutaminolysis/TCA intermediates (Glu and DM-αKG) did not restore BT142 cell proliferation in the presence of JHU-083 (Figure 3C). GSH and Glu moderately rescue cell proliferation in U251 cell in glutamine-deprived media (Supplementary Figure 3C). To test if the oncometabolite 2HG is essential for JHU-083 antiproliferative effect, we treated BT142 *IDH1R132H* mutant cells with the IDH1R132H inhibitor, AGI-5198, in combination with JHU-083. The combination of JHU-083 with AGI-5198 did not further modulate cell growth in BT142 cells. Additionally, it has been shown that targeting glutamine metabolism activates autophagy. Therefore, we used Chloroquine, a late-stage autophagy inhibitor, in combination with JHU-083. Chloroquine treatment did not further modulate cell proliferation in the presence of JHU-083 (Figure 3C). Finally, nonessential amino acids (in combination or in high concentration individually) did not restore cell proliferation reduction induced by JHU-083 (Supplementary Figure 3A and B).

### JHU-083 Disrupts mTOR Signaling Pathway and Reduces Cyclin D1 Expression

mTOR is a serine/threonine kinase highly conserved among species and functions in 2 structural distinct protein complexes, named mTORC1 and mTORC2. mTORC1 integrates growth factor signaling and nutrient levels, acting as an intracellular nutrient sensor, while mTORC2 phosphorylate AGC kinases, such as AKT(ser473), in response to growth factors. The best characterized downstream effectors of mTORC1 are p70 S6 kinase and eukaryotic initiation factor (eIF)4E-binding protein (4E-BP), and pAKT(ser473) for mTORC2. These mTOR downstream pathways have been implicated in brain cancers.^19^ The interaction between glutamine metabolism and mTOR signaling is mediated by αKG generated from glutaminolysis and it supports mTORC1 lysosomal translocation and activation.^20^ The observation that JHU-083 reduced Glu levels representing a potential regulation of mTOR signaling through glutaminolysis.

**Figure 4.**
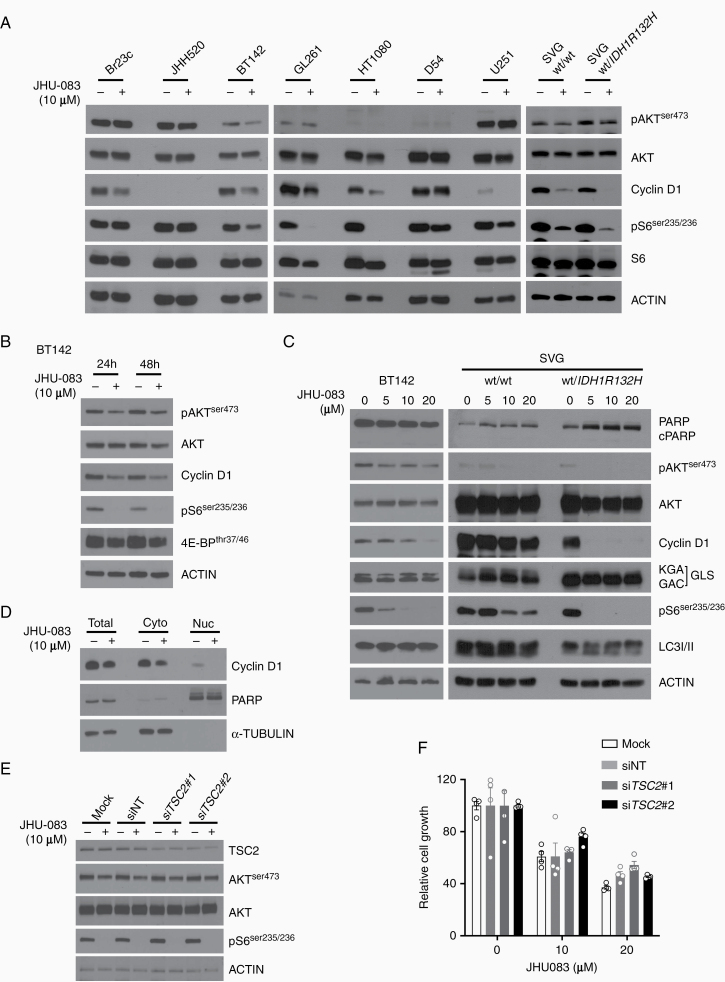
mTOR signaling pathway disruption and Cyclin D1 protein expression deregulation in JHU-083-treated cells. (A and B) Protein expression was analyzed in cancer cells were treated with JHU-083. (C) Protein expression was analyzed in cells treated with JHU-083 at indicated concentration (24 h). (D) Cytoplasmic and nuclear fractions were analyzed in BT142 treated with JHU-083 (24 h). (E and F) BT142 cells were transfected with siRNA targeting *TSC2*, treated with JHU-083 and protein expression (48 h) and cell viability (72 h) were analyzed.

To further explore the impact of JHU-083 in signaling pathways associated with glutamine metabolism, cancer cell lines and SVG (wt/wt) and SVG (wt/*IDH1R132H*) were treated with JHU-083. Cyclin D1 protein expression was reduced and correlated with pS6(Ser235/236) downregulation in 5 of 7 cancer cell lines treated with JHU-083. pAKT(ser473) expression were slightly downregulated in some cell lines. SVG (wt/*IDH1R132H*) showed preferential reduction in pS6(Ser235/236) and Cyclin D1 when compared with SVG (wt/wt) (Figure 4A). Time-course JHU-083 treatment showed consistent Cyclin D1, pS6(ser235/236), 4E-BP(thr37/46) protein expression reduction, and a slight decrease in pAKT(ser473) expression in BT142 cells (Figure 4B). JAM326 showed similar reduction in pS6(ser235/236) protein expression in BT142 cells (Supplementary Figure 4A). Cells cultivated in media supplemented with Glu did not rescue pS6(ser235/236) and Cyclin D1 downregulation induced by JHU-083 (Supplementary Figure 4B). Dose-dependent reduction was observed in Cyclin D1, pS6(ser235/236), but SVG (wt/*IDH1R132H*) preferentially reduced those proteins when compared with parental cells. pAKT(ser473) protein expression was consistently reduced in BT142 (Figure 4C). In addition, cleaved-PARP and LC3I/II were not modulated by JHU-083, suggesting no impact in apoptosis and autophagy-associated cell death, respectively. Long-term JHU-083 exposure did not modulate cleaved-PARP protein expression (Supplementary Figure 4C). To characterize Cyclin D1 localization, cytosolic and nuclear fractions from BT142 cells treated with JHU-083 were collected and Cyclin D1 subcellular location protein expression was analyzed. JHU-083 reduced total and cytoplasmic Cyclin D1 protein expression and completely abolished Cyclin D1 localization in the nucleus (Figure 4D).

A central node in the mTOR signaling pathway is the TSC1/2 complex, which integrates growth factors signaling, cellular stress, and energy levels with mTOR signaling. TSC2 is a GTPase-activating protein upstream of mTOR protein complex that negatively modulates mTORC1 signaling at the lysosomal level.^21^ To obtain insight on how growth factors and others mTOR upstream factors impact cell proliferation by JHU-083, we silenced the *TSC2* gene and analyzed cell proliferation and mTOR signaling. *TSC2* gene silencing was confirmed by TSC2 protein expression reduction after si*TSC2* transfection in BT142 cells. JHU-083 treatment, as expected, reduced pS6(ser235/236) and slightly reduced pAKT(ser473) in BT142 cells. Interestingly, JHU-083 showed similar protein expression (pS6(ser235/236) and pAKT(ser473)) effect in *TSC2* gene silencing and controls (mock and non-target siRNA) (Figure 4E). Furthermore, *TSC2* gene silencing did not modulate growth inhibition induced by JHU-083 in BT142 cells (Figure 4F).

### Guanine Partially Restores JHU-083 Antiproliferative Effect and mTOR Signaling

**Figure 5.**
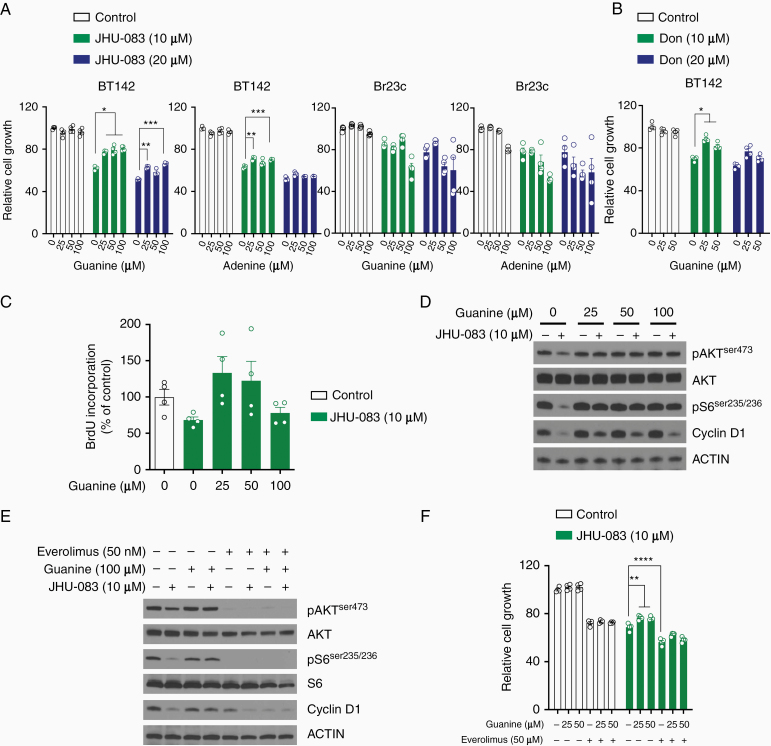
Cell proliferation, DNA synthesis, and mTOR signaling pathway are partially restored in cells cotreated with JHU-083 and guanine. (A) Cell viability was performed in cells treated with JHU-083 in combination with guanine or adenine (96 h). (B) Cell viability was performed in cells treated with Don in combination with guanine (96 h). (C) BrdU incorporation in BT142 treated with JHU-083 in combination with guanine (24 h). (D) Protein expression in BT142 treated with JHU-083 and guanine (24 h). (E) Cells were treated with JHU-083 (24 h) in combination with guanine and/or everolimus at indicated concentration and protein expression was analyzed by Western blot. (F) Cell viability was performed in BT142 treated with JHU-083 in combination with guanine and everolimus (96 h). *P < .05; **P < .01; ***P < .001; ****P < .0001 versus respective control.

Glutamine is essential to de novo purine and pyrimidine synthesis through γ-nitrogen donation. In glioblastoma stem-like cells, carbons derived from glucose are used in de novo purine synthesis pathway. In addition, enzymes associated with purine biosynthesis pathway are upregulated in glioblastoma stem-like cells.^22^ It has been shown that DON can directly inhibit glutamine amidotransferase activity and compromise de novo nucleotide synthesis.^6^ Therefore, considering glutaminolysis had small impact on JHU-083-mediated cell proliferation inhibition, it was reasonable to test whether guanine and adenine impact cell proliferation. Guanine treatment partially rescued the JHU-083 antiproliferative effect in BT142 cells (*IDH*mut), but not in Br23c cell line (*IDH* wild type) (Figure 5A). Additionally, BT142 cells supplemented with guanine in the presence of DON partially restored cell proliferation (Figure 5B). Adenine showed small cell proliferation recovery in BT142 cells treated with JHU-083. To complement the cell proliferation assay, we measured DNA synthesis by BrdU incorporation in this condition (Figure 5C). Guanine at different concentrations was found to restore pS6(ser235/236), pAKT(ser473), and Cyclin D1 protein expression in BT142 cells cotreated with JHU-083 (Figure 5D). The combination of guanine with adenine or DM-αKG in cells treated with JHU-083 did not further rescue cell proliferation (Supplementary Figure 5). These data suggest that guanine abrogates JHU-083-mediated cell proliferation reduction and mTOR signaling downregulation. Based on this model proposed and the data acquired in Figure 5A–D, we wondered whether the effect of cell proliferation inhibition induced by JHU-083 is dependent on mTOR signaling. Cells treated with JHU-083 and guanine in the presence of mTOR inhibitor (everolimus) reduced pAKT(ser473) and pS6(ser235/236) and further reduced Cyclin D1 protein expression (Figure 5E). Everolimus as single agent reduced cell proliferation and this effect was enhanced in combination with JHU-083. Guanine in the presence of JHU-083 and everolimus did not restore cell proliferation (Figure 5F). Taken together, these results demonstrate the requirement of mTORC1 signaling activation to recovery cell proliferation induced by guanine in cells treated with JHU-083.

### JHU-083 Treatment Prolongs Survival of Orthotopic IDH1R132H Glioma Model

**Figure 6.**
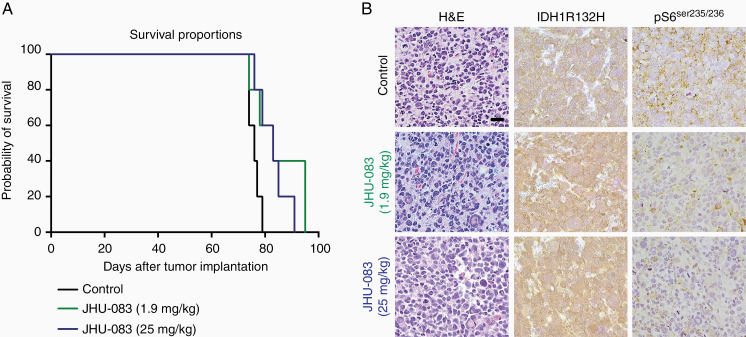
JHU-083 extends survival in orthotopic *IDH1R132H* glioma. (A) Kaplan–Meier survival curve of BT142 in orthotopic xenograft treated with vehicle solution or JHU-083 (1.9 mg/kg or 25 mg/kg; *n* = 5 group). (B) Representative images of H&E, IDH1R132H, and pS6(ser235/236) immunohistochemistry. Scale bar 50 µm.

We next evaluated if targeting glutamine metabolism with JHU-083 could extend survival in an orthotopic glioma model. It has been shown that JHU-083 prodrug efficiently delivers DON into the brain.^8,18^ Therefore, BT142 *IDH1R132H* glioma cells were implanted in nude mice to evaluate the efficacy of chronic administration of JHU-083 in vivo. At the lower dose of JHU-083 tested (1.9 mg/kg) survival benefit was not apparent (*P* = .053 vs control), but the higher JHU-083 dose (25 mg/kg) improved survival (*P* = .027 vs control; Figure 6A). Daily low doses of JHU-083 decreased mice body mass, but recovery was observed when JHU-083 was administrated at a lower frequency (2×/week). No toxicity was observed with higher JHU-083 (2×/week) (Supplementary Figure 6). Histological evaluation of orthotopic BT142 from mice treated with JHU-083 showed strong IDH1R132H protein expression and reduced pS6(ser235/236) staining (Figure 6B), suggesting that JHU-083 reaches intracranial cells and targets mTORC1 signaling pathway in vivo.

## Discussion

Cancer cells rely upon a metabolic reprogramming, with high glycolytic flux in normoxic conditions and a high rate of glutaminolysis. Glutamine is a donor of γ-nitrogen to purine and pyrimidine biosynthesis, and contributes to TCA cycle anaplerosis, lipid and protein synthesis, and redox balance.^5^  *IDH*mut glioma showed deregulated glutamine metabolism with more reliance on glutamine utilization.^11,12^ Here, we showed that JHU-083, a glutamine antagonist prodrug, reduces cell proliferation in molecularly diverse cancer cells and decreases mTOR signaling. Mechanistically guanine nucleobase treatment was found to partially restore cell proliferation, DNA synthesis, and mTOR signaling downregulation in vitro in cells treated with JHU-083. Importantly, we show that JHU-083 extends survival in orthotopic *IDH1*mut glioma model, through targeting mTORC1 signaling pathway and cell growth inhibition.

The precise biological role of each GLS isoforms (KGA and GAC) and GLS2 gene in brain cancer is not completely understood. siRNA-mediated *GLS* gene silencing or *GLS2* overexpression reduces glioblastoma cell growth in vitro.^23^ Modest growth inhibition in glioma cells was observed with *GLS* gene silencing, likely due to the incomplete mitochondrial GAC isoform silencing that compensates the almost complete KGA isoform silencing. *GLS* gene silencing or allosteric inhibition by pharmacological compounds is not directly comparable with glutamine deprivation because glutamine participates in other reactions than the generation of Glu catalyzed by GLS enzyme. In contrast with *GLS* gene silencing, glutamine deprivation noticeably reduces cell growth and it has been shown to be associated with intrinsic apoptosis activation.^24^ Although glutamine-free cell culture conditions are a consistent tool to evaluate the role of exogenous glutamine metabolism in cancer biology in vitro, strategies to target glutamine metabolism specifically in glioma cells in vivo are lacking. On the other hand, targeting glutamine metabolism with broad glutamine antagonists or GLS inhibitors are currently available and they have demonstrated preclinical efficacy in several cancer types.^8,11^ The pleiotropic effect of a glutamine antagonist provides a possible opportunity to disrupt the glutamine metabolism addiction observed of malignant growth. We observed that JHU-083 induces growth inhibition in cancer cells irrespectively of *IDH1/2*mut status, likely due to additional intrinsic genetic and molecular differences between cell lines evaluated in our study. However, isogenic human normal astrocyte (SVG cells) with heterozygous *IDH1R132H* mutation and D54 glioma cell with doxycycline-inducible IDH1R132H protein showed reduced cell proliferation in the presence of JHU-083. In addition, it has been shown that allosteric inhibition of GLS with BPTES or CB-839 preferentially decreases *IDH1*mut isogenic glioma and AML cells.^11,12,16,25,26^

JHU-083 can deliver DON to the brain at micromolar concentrations achieving therapeutic levels after systemic administration in mouse, swine, and monkey models.^8,14,27^ The growth reduction observed after JHU-083 is likely associated with G0/G1 cell cycle arrest by decreased mTORC1 activity and Cyclin D1 protein expression reduction. mTORC1 signaling regulates Cyclin D1 protein expression through a mechanism independent of gene transcription and directly regulates cell cycle progression from G0/G1 to S phase.^28^ Cyclin D1 is overexpressed in multiple cancer types, including glioma. Importantly, Cyclin D1 which is downregulated in the nucleus, controls the transition of G0/G1 cell cycle phase to S phase and it is correlated with lower DNA synthesis and cell proliferation after JHU-083 treatment. Based on our data, it is tempting to speculate that the downregulation of Cyclin D1 protein expression/location and cell proliferation inhibition are both directly associated with mTOR signaling disruption induced by JHU-083. Changing the threshold for cell cycle phase transition and delaying DNA replication are critical steps for cancer cell growth control and this approach has been recently exploited with cell cycle modulation inhibitors for cancer therapy.^29^

There are mixed results determining the effect of an *IDH1R132H* mutation on mTOR signaling. *IDH1R132H* mutation activates the mTOR signaling pathway and pharmacological inhibition of IDH1R132H by AGI-5198 reduces (*R*)-2HG and decreases mTOR signaling.^30^ However, glioma cells overexpressing *IDH1R132H* or parental cells treated with (*R*)-2HG inhibit mTORC1 signaling in an AMPK-dependent manner.^31^ mTORC1 can be regulated by glutaminolysis, and other amino acids, for example, leucine through distinct mechanisms,^20,32^ and through intracellular purine and pyrimidine nucleotide levels.^33–35^ Recently, it has been shown that purine nucleotide levels can inhibit mTOR signaling through Rheb protein expression downregulation or post-translational modifications.^33,36^ Conversely, mTOR can positively regulate de novo pyrimidine and purine synthesis,^34,35^ suggesting a bidirectional regulation between nucleotide levels and mTOR signaling stimulating nucleotide synthesis. Interestingly, cancer cells supplemented with exogenous nucleotides, but not with intermediates of TCA, rescue to some extent cell growth in glutamine-deprived condition.^10^ Collectively, these data suggest that mTOR integrates growth factor signaling with amino acids and nucleotide levels in a context-dependent mechanism. Although we did not design our experiments to directly compare the relative contribution of amino acids and nucleotides to mTOR signaling, we believe that the main mechanism of mTOR regulation in the presence of JHU-083 is through purine de novo biosynthesis. DON inhibits enzymes associated with purine biosynthesis and reduction in this pathway inhibits mTORC1 signaling independently of the energy status and AMPK signaling.^36^ Conversely, guanine nucleobase supplementation, but not TCA cycle intermediates and nonessential amino acids, recover to some extent cell proliferation, DNA synthesis, and mTORC1 inhibition induced by JHU-083 exposure, supporting that mTOR signaling and de novo purine synthesis are the main pathway disrupted by JHU-083 in vitro.

JHU-083 showed a significant survival benefit in an orthotopic *IDH1*mut glioma model, highlighting the importance of glutamine metabolism for in vivo growth. The limited effect observed in mice treated with JHU-083 is perhaps determined by the ability of cells in the microenvironment to synthesize and release purine nucleobase and other related nucleotides. Indeed, recently, this was described as a mechanism of gemcitabine, a deoxycytidine analogue, resistance in pancreatic ductal adenocarcinoma. Tumor-associated macrophages release pyrimidine to the microenvironment driving gemcitabine resistance in a paracrine fashion into cancer cells via competition with gemcitabine uptake and metabolism.^37^ In addition, we should take into account that glioma cells can compensate disrupted pathways and metabolites/substrates limitation by activation of additional energy sources.^24^ Because cell division and appropriate intracellular carbon and nitrogen precursors levels are tightly connected in cancer cells, disturbing both mTOR signaling and glutamine metabolism is a clinically significant strategy. Regardless of JHU-083 efficacy in *IDH1*mut model, this prodrug, with brain penetrant properties and known intracellular targets, could be used as a platform for future drug combinations. In fact, few therapies are used as single agents to treat cancer, and additional drug combination could improve survival with tolerable toxic effect in normal cells. Additional preclinical evidence suggests the utility of broad glutamine antagonism in cancer therapy. Recently, orally administrated JHU-083 extended survival in vivo in orthotopic MYC-amplified medulloblastoma models.^8^ Another oral glutamine antagonist prodrug, termed JHU395, reduced malignant peripheral nerve sheath tumor through purine synthesis inhibition with no apparent toxicity in vivo.^9^  *IDH1R132H* mutant cells bear dependence to glutamine metabolism through BCAT1 and BCAT2 enzyme activity inhibition and consequently Glu reduction levels, a mechanism that explains the high Glu-dependent production by glutaminolysis in *IDH1*mut gliomas.^12^ Targeting *IDH1R132H* mutant glioma with the glutaminase inhibitor CB-389 showed cell growth reduction in vitro, but not in vivo as single agent. However, combination with radiation extends survival, suggesting that further drug combinations are needed to optimize GLS inhibitors for glioma therapy.^12^

Targeting metabolism for brain and other cancer therapy has garnered recent interest and research. Improving drugs with known metabolic targets is a reasonable strategy to evaluate for potential metabolic therapies. The antiproliferative effect of JHU-083 is mediated by a mechanism that is associated with reprogramming of glutamine metabolism, which in turn deregulates purine metabolism, and mTORC1 signaling. These data are of great importance because several clinical trials are currently testing GLS inhibitors in solid cancers. In conclusion, the administration of JHU-083 was tolerated and extended survival in orthotopic *IDH1R132H* mutant model, likely due the cell cycle arrest and mTOR signaling pathway inhibition. Taken together, these data support the current concept of targeting glutamine metabolism in cancer. This study highlights the essential role of glutamine metabolism in glioblastoma cells and provides a potential therapeutic opportunity to target glutamine metabolism in *IDH1/2* mutant cancers.

## Supplementary Material

vdaa149_suppl_Supplementary_Figures_S1-S7

vdaa149_suppl_Supplementary_Materials

## Notes

**Conflict of interest statement.** B.S.S. and R.R. are scientific founders and hold equity in Dracen Pharmaceuticals, a company pursuing small molecule glutamine antagonists for clinical oncology applications. Glutamine antagonists, including JHU083, were patented by B.S.S. and R.R. and Johns Hopkins University and subsequently licensed to Dracen Pharmaceuticals. Other authors declare no conflicts of interest.

## Notes

**Authorship Statement.** A.S.Y., M.C.R., and G.J.R. conceived, designed, and development methodologies. A.S.Y. and M.C.R. carried out experiments. V.S. performed gene expression analysis. R.R. and B.S. provided the glutamine antagonists, dosing, stability, and formulation information. A.S.Y., M.C.R., and G.J.R. analyzed, interpreted data, and wrote the paper. G.J.R. supervised the study.

## References

[1] Parsons DW, Jones S, Zhang X, et al. An integrated genomic analysis of human glioblastoma multiforme. Science. 2008;321(5897): 1807–1812. 18772396 10.1126/science.1164382 PMC2820389

[2] Waitkus MS, Diplas BH, Yan H. Isocitrate dehydrogenase mutations in gliomas. Neuro Oncol. 2016;18(1):16–26. 26188014 10.1093/neuonc/nov136 PMC4677412

[3] Louis DN, Perry A, Reifenberger G, et al. The 2016 World health organization classification of tumors of the central nervous system: a summary. Acta Neuropathol. 2016;131(6):803–820. 27157931 10.1007/s00401-016-1545-1

[4] Noushmehr H, Weisenberger DJ, Diefes K, et al. Identification of a CpG island methylator phenotype that defines a distinct subgroup of glioma. Cancer Cell. 2010;17(5):510–522. 20399149 10.1016/j.ccr.2010.03.017 PMC2872684

[5] Zhang J, Pavlova NN, Thompson CB. Cancer cell metabolism: the essential role of the nonessential amino acid, glutamine. EMBO J. 2017;36(10):1302–1315. 28420743 10.15252/embj.201696151 PMC5430235

[6] Lemberg KM, Vornov JJ, Rais R, Slusher BS. We’re Not “DON” Yet: optimal dosing and prodrug delivery of 6-Diazo-5-oxo-L-norleucine. Mol Cancer Ther. 2018;17(9):1824–1832. 30181331 10.1158/1535-7163.MCT-17-1148 PMC6130910

[7] Tanaka K, Sasayama T, Irino Y, et al. Compensatory glutamine metabolism promotes glioblastoma resistance to mTOR inhibitor treatment. J Clin Invest. 2015;125(4):1591–1602. 25798620 10.1172/JCI78239 PMC4396477

[8] Hanaford AR, Alt J, Rais R, et al. Orally bioavailable glutamine antagonist prodrug JHU-083 penetrates mouse brain and suppresses the growth of MYC-driven medulloblastoma. Transl Oncol. 2019;12(10):1314–1322. 31340195 10.1016/j.tranon.2019.05.013 PMC6657308

[9] Lemberg KM, Zhao L, Wu Y, et al. The novel glutamine antagonist prodrug JHU395 has antitumor activity in malignant peripheral nerve sheath tumor. Mol Cancer Ther. 2020;19(2):397–408. 31594823 10.1158/1535-7163.MCT-19-0319 PMC7007868

[10] Tardito S, Oudin A, Ahmed SU, et al. Glutamine synthetase activity fuels nucleotide biosynthesis and supports growth of glutamine-restricted glioblastoma. Nat Cell Biol. 2015;17(12):1556–1568. 26595383 10.1038/ncb3272 PMC4663685

[11] Seltzer MJ, Bennett BD, Joshi AD, et al. Inhibition of glutaminase preferentially slows growth of glioma cells with mutant IDH1. Cancer Res. 2010;70(22):8981–8987. 21045145 10.1158/0008-5472.CAN-10-1666 PMC3058858

[12] McBrayer SK, Mayers JR, DiNatale GJ, et al. Transaminase inhibition by 2-hydroxyglutarate impairs glutamate biosynthesis and redox homeostasis in glioma. Cell. 2018;175(1):101–116 e125. 30220459 10.1016/j.cell.2018.08.038 PMC6219629

[13] Yamashita AS, da Costa Rosa M, Borodovsky A, Festuccia WT, Chan T, Riggins GJ. Demethylation and epigenetic modification with 5-azacytidine reduces IDH1 mutant glioma growth in combination with temozolomide. Neuro Oncol. 2019;21(2):189–200. 30184215 10.1093/neuonc/noy146 PMC6374765

[14] Rais R, Jančařík A, Tenora L, et al. Discovery of 6-Diazo-5-oxo-l-norleucine (DON) prodrugs with enhanced CSF delivery in monkeys: a potential treatment for glioblastoma. J Med Chem. 2016;59(18):8621–8633. 27560860 10.1021/acs.jmedchem.6b01069

[15] Borodovsky A, Meeker AK, Kirkness EF, et al. A model of a patient-derived IDH1 mutant anaplastic astrocytoma with alternative lengthening of telomeres. J Neurooncol. 2015;121(3):479–487. 25471051 10.1007/s11060-014-1672-2 PMC4648276

[16] Matre P, Velez J, Jacamo R, et al. Inhibiting glutaminase in acute myeloid leukemia: metabolic dependency of selected AML subtypes. Oncotarget. 2016;7(48):79722–79735. 27806325 10.18632/oncotarget.12944 PMC5340236

[17] Wei S, Wang J, Oyinlade O, et al. Heterozygous IDH1R132H/WT created by “single base editing” inhibits human astroglial cell growth by downregulating YAP. Oncogene. 2018;37(38):5160–5174. 29849122 10.1038/s41388-018-0334-9 PMC6590918

[18] Zhu X, Nedelcovych MT, Thomas AG, et al. JHU-083 selectively blocks glutaminase activity in brain CD11b+ cells and prevents depression-associated behaviors induced by chronic social defeat stress. Neuropsychopharmacology. 2019;44(4):683–694. 30127344 10.1038/s41386-018-0177-7 PMC6372721

[19] Pachow D, Wick W, Gutmann DH, Mawrin C. The mTOR signaling pathway as a treatment target for intracranial neoplasms. Neuro Oncol. 2015;17(2):189–199. 25165193 10.1093/neuonc/nou164 PMC4288522

[20] Durán RV, Oppliger W, Robitaille AM, et al. Glutaminolysis activates Rag-mTORC1 signaling. Mol Cell. 2012;47(3):349–358. 22749528 10.1016/j.molcel.2012.05.043

[21] Menon S, Dibble CC, Talbott G, et al. Spatial control of the TSC complex integrates insulin and nutrient regulation of mTORC1 at the lysosome. Cell. 2014;156(4):771–785. 24529379 10.1016/j.cell.2013.11.049 PMC4030681

[22] Wang X, Yang K, Xie Q, et al. Purine synthesis promotes maintenance of brain tumor initiating cells in glioma. Nat Neurosci. 2017;20(5):661–673. 28346452 10.1038/nn.4537 PMC6015494

[23] Szeliga M, Bogacińska-Karaś M, Różycka A, Hilgier W, Marquez J, Albrecht J. Silencing of GLS and overexpression of GLS2 genes cooperate in decreasing the proliferation and viability of glioblastoma cells. Tumour Biol. 2014;35(3):1855–1862. 24096582 10.1007/s13277-013-1247-4 PMC3967065

[24] Zhang J, Fan J, Venneti S, et al. Asparagine plays a critical role in regulating cellular adaptation to glutamine depletion. Mol Cell. 2014;56(2):205–218. 25242145 10.1016/j.molcel.2014.08.018 PMC4224619

[25] Ohka F, Ito M, Ranjit M, et al. Quantitative metabolome analysis profiles activation of glutaminolysis in glioma with IDH1 mutation. Tumour Biol. 2014;35(6):5911–5920. 24590270 10.1007/s13277-014-1784-5

[26] Emadi A, Jun SA, Tsukamoto T, Fathi AT, Minden MD, Dang CV. Inhibition of glutaminase selectively suppresses the growth of primary acute myeloid leukemia cells with IDH mutations. Exp Hematol. 2014;42(4):247–251. 24333121 10.1016/j.exphem.2013.12.001

[27] Nedelcovych MT, Tenora L, Kim BH, et al. N-(Pivaloyloxy)alkoxy-carbonyl prodrugs of the glutamine antagonist 6-diazo-5-oxo-l-norleucine (DON) as a potential treatment for HIV associated neurocognitive disorders. J Med Chem. 2017;60(16):7186–7198. 28759224 10.1021/acs.jmedchem.7b00966 PMC5795620

[28] Fingar DC, Richardson CJ, Tee AR, Cheatham L, Tsou C, Blenis J. mTOR controls cell cycle progression through its cell growth effectors S6K1 and 4E-BP1/eukaryotic translation initiation factor 4E. Mol Cell Biol. 2004;24(1):200–216. 14673156 10.1128/MCB.24.1.200-216.2004 PMC303352

[29] Sherr CJBJ. Cell cycle-targeted cancer therapies. Annu Rev Cancer Biol. 2017;1:16.

[30] Carbonneau M, Gagné LM, Lalonde ME, et al. The oncometabolite 2-hydroxyglutarate activates the mTOR signalling pathway. Nat Commun. 2016;7:12700. 27624942 10.1038/ncomms12700 PMC5027283

[31] Fu X, Chin RM, Vergnes L, et al. 2-Hydroxyglutarate inhibits ATP synthase and mTOR signaling. Cell Metab. 2015;22(3):508–515. 26190651 10.1016/j.cmet.2015.06.009 PMC4663076

[32] Jewell JL, Kim YC, Russell RC, et al. Metabolism. Differential regulation of mTORC1 by leucine and glutamine. Science. 2015;347(6218):194–198. 25567907 10.1126/science.1259472 PMC4384888

[33] Emmanuel N, Ragunathan S, Shan Q, et al. Purine nucleotide availability regulates mTORC1 activity through the Rheb GTPase. Cell Rep. 2017;19(13):2665–2680. 28658616 10.1016/j.celrep.2017.05.043

[34] Ben-Sahra I, Howell JJ, Asara JM, Manning BD. Stimulation of de novo pyrimidine synthesis by growth signaling through mTOR and S6K1. Science. 2013;339(6125):1323–1328. 23429703 10.1126/science.1228792 PMC3753690

[35] Robitaille AM, Christen S, Shimobayashi M, et al. Quantitative phosphoproteomics reveal mTORC1 activates de novo pyrimidine synthesis. Science. 2013;339(6125):1320–1323. 23429704 10.1126/science.1228771

[36] Hoxhaj G, Hughes-Hallett J, Timson RC, et al. The mTORC1 signaling network senses changes in cellular purine nucleotide levels. Cell Rep. 2017;21(5):1331–1346. 29091770 10.1016/j.celrep.2017.10.029 PMC5689476

[37] Halbrook CJ, Pontious C, Kovalenko I, et al. Macrophage-released pyrimidines inhibit gemcitabine therapy in pancreatic cancer. Cell Metab. 2019;29(6):1390–1399 e1396. 30827862 10.1016/j.cmet.2019.02.001 PMC6602533

