# Supplementary material for: The glutamine antagonist prodrug JHU-083 slows malignant glioma growth and disrupts mTOR signaling
Source: Neurooncol Adv. 2020 Oct 29;3(1):vdaa149. doi: 10.1093/noajnl/vdaa149 (PMC7920530; doi:10.1093/noajnl/vdaa149)

Supplementary Figure 1

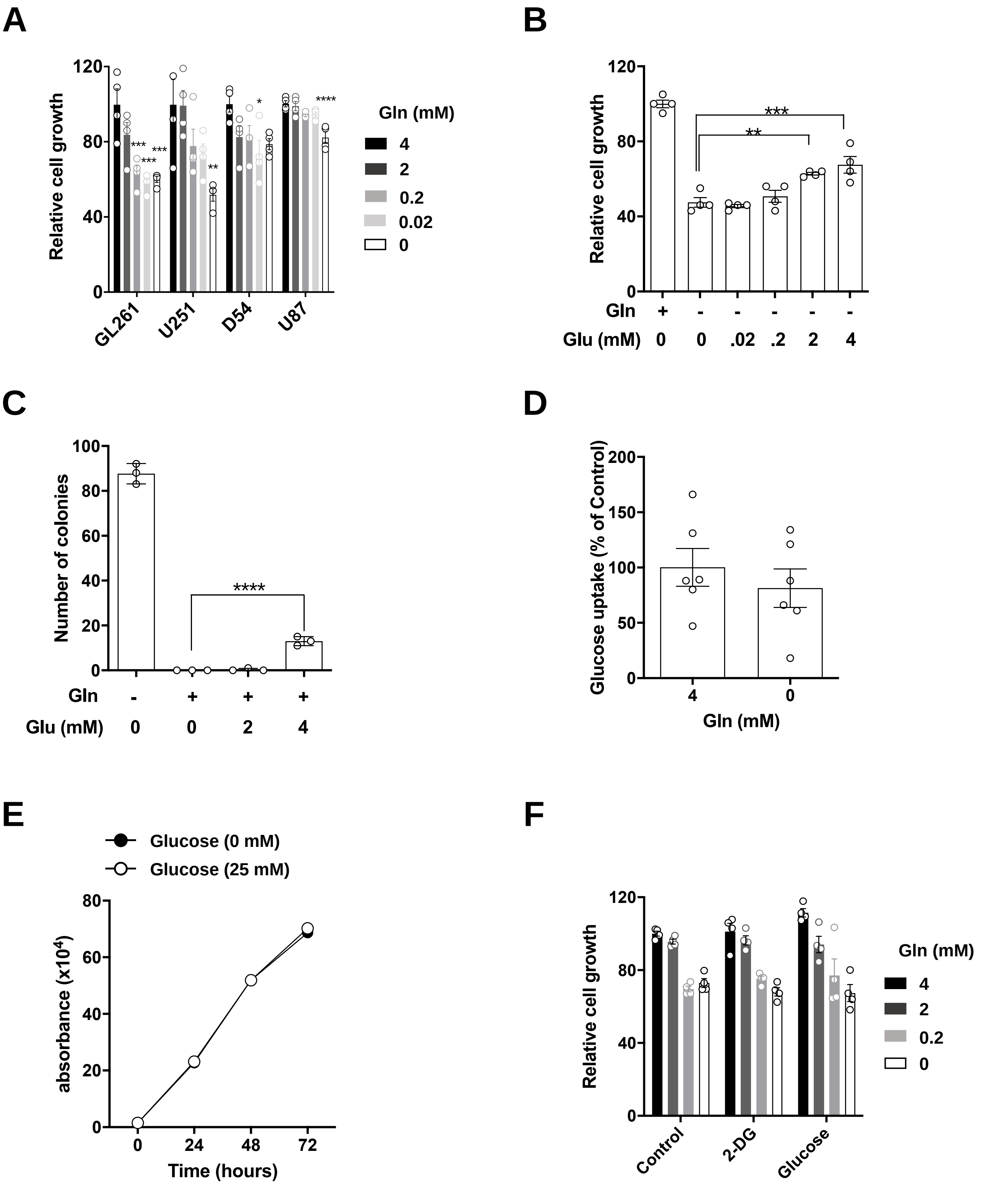

Supplementary Figure 2

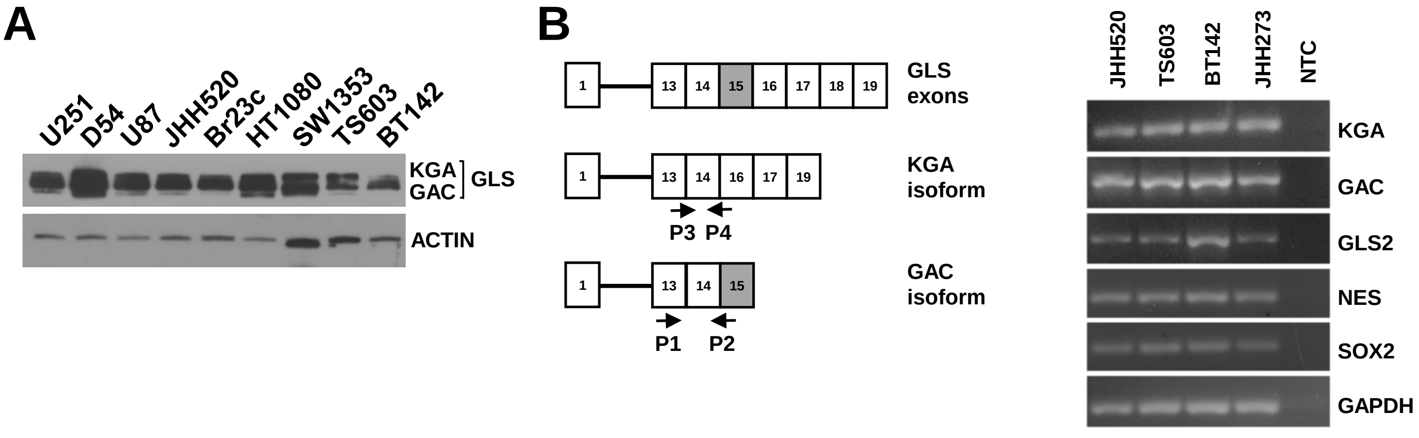

Supplementary Figure 3

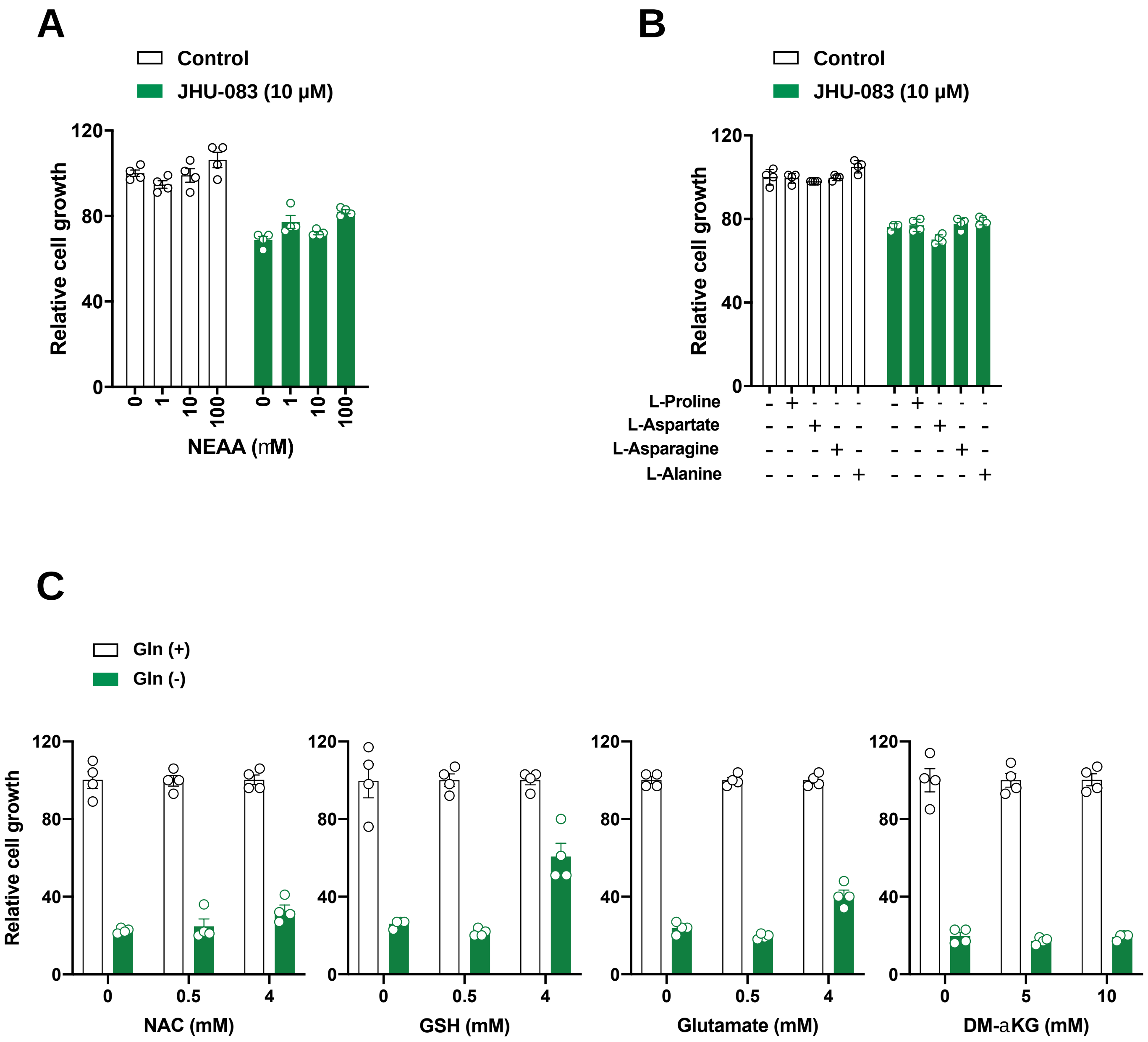

Supplementary Figure 4

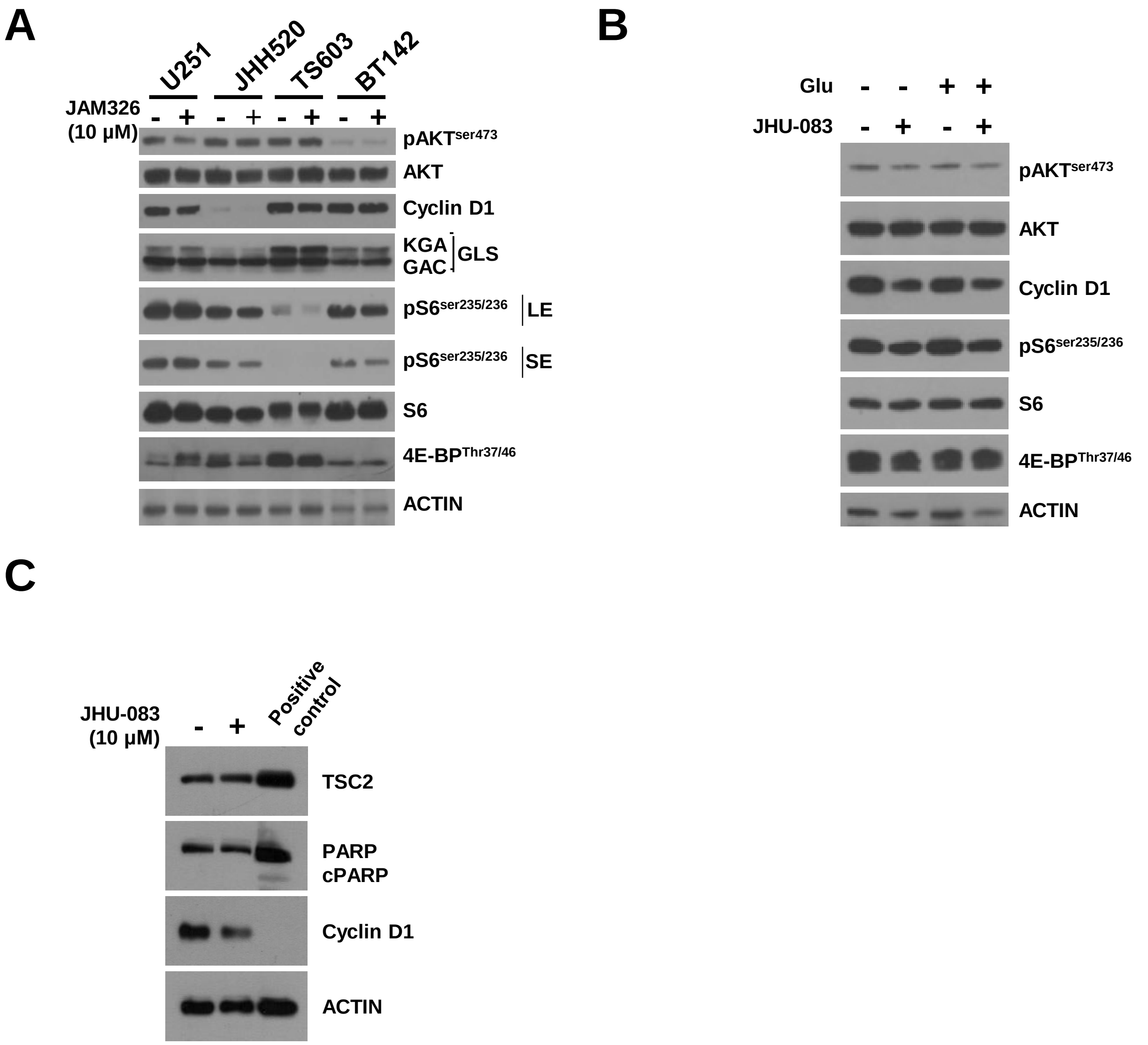

Supplementary Figure 5

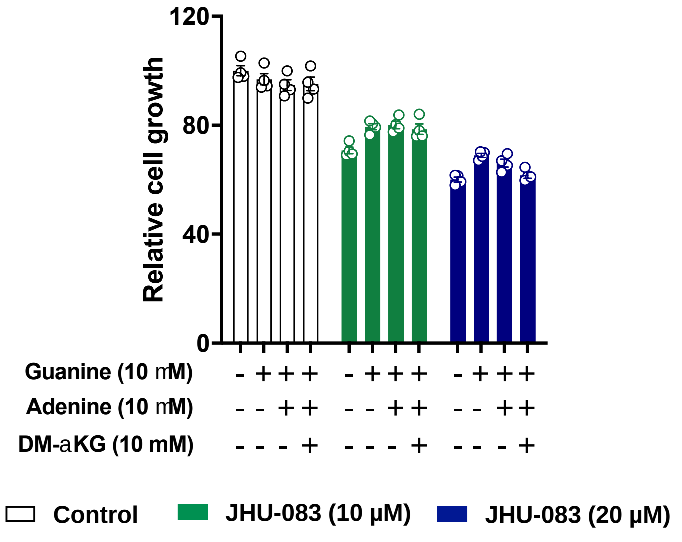

Supplementary Figure 6

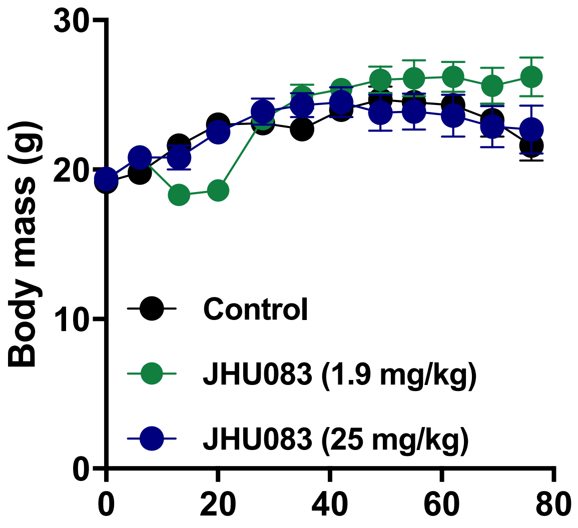

Supplementary Figure 7

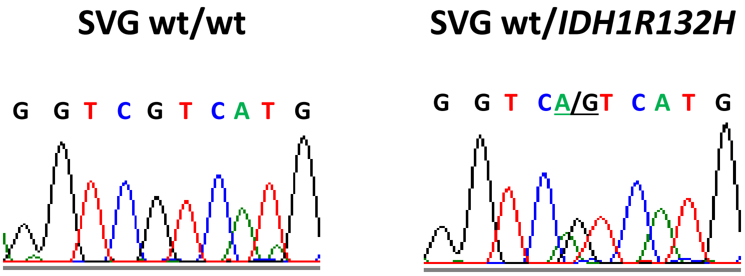

Supplement: vdaa149_suppl_Supplementary_Figures_S1-S7 [file vdaa149_suppl_supplementary_figures_s1-s7.pdf]
