# Supplementary material for: The glutamine antagonist prodrug JHU-083 slows malignant glioma growth and disrupts mTOR signaling
Source: Neurooncol Adv. 2020 Oct 29;3(1):vdaa149. doi: 10.1093/noajnl/vdaa149 (PMC7920530; doi:10.1093/noajnl/vdaa149)
Supplement: vdaa149_suppl_Supplementary_Materials [file vdaa149_suppl_supplementary_materials.docx]

**SUPPLEMENTARY INFORMATION**

**SUPPLEMENTARY MATERIAL AND METHODS**

*Cell culture and reagents*

Human GBM U87 MG, U251 MG, D54 MG, (hereafter referred to U87, U251, D54, respectively), D54 doxycycline-inducible IDH1R132H expression, SVG parental (wt/wt), SVG (wt/IDHR132H), mouse GBM GL261, human chondrosarcoma SW1353, human fibrosarcoma HT1080, and HEK29T cells were maintained as monolayer in Dulbecco’s Modified Eagle Media (DMEM) (11965-092 - Gibco, Thermo Fisher Scientific) supplemented with 10% Fetal Bovine Serum (Gemini Bio-Products). BT142, TS603, JHH520, and Br23C were cultured with Neurobasal serum-free media supplemented with 10 ng/mL Fibroblast Growth Factor (bFGF), 20 ng/mL Epidermal Growth Factor (EGF) (Peprotech), and 4 µg/mL heparin (Sigma). BT142, SW1353, and HT1080 were purchased from ATCC. TS603 was kindly provided by Dr. Timothy Chan (Memorial Sloan Kettering Cancer Center, New York, NY). JHH520, Br23C, and JHH273 were established in our laboratory ^1,2^. IDH1R132H protein expression was induced with doxycycline (Doxy - 40 ng/mL) in D54 cell line as previous described ^3^. IDH1R132H expression was induced with Doxy 48 hours before JHU-083 treatment in cell proliferation analysis. Human astroglial SVG cell line with monoallelic IDH1R132H generated by single base editing was kindly donated by Dr. Shuli Xia (Johns Hopkins University, Baltimore, MD) ^4^. Heterozygous IDH1R132H mutation was confirmed by Sanger DNA sequencing as previous described ^5^. Supplementary figure 7 shows sequencing chromatograms confirming the heterozygous *IDH1R132H* mutation in SVG (wt/IDH1R132H) cells. All cells were maintained in a humidified incubator at 5 % CO_2_ and 37 ºC. Cells were tested to for mycoplasma using MycoAlert Mycoplasma Detection Kit following manufacture’s instruction (Lonza). U251, BT142, JHH520, and Br23c were authenticated by STR profile at Johns Hopkins Genomic Core facility. Constitutive *GLS* gene silencing was performed with pLKO.1 (sh*GLS*#1 and sh*GLS*#2 with TRCN0000051133 and TRCN0000051134, respectively) as previously described ^6^ and maintained with Puromycin (1 µg/mL) (Sigma). Two independent small interfering RNA (siRNA) (30 nM) were used to silence *GLS* (si*GLS*#1 and si*GLS*#2 with hs.Ri.GLS.13.2 and hs.Ri.GLS.13.1, respectively) and *TSC2* (si*TSC2*#1 and si*TSC*2#2 with hs.Ri.TSC2.13.2 and hs.Ri.TSC2.13.2, respectively) (Integrated DNA Technology - IDT). Non-target siRNA control (IDT) and non-transfected cells (Mock control) were used as controls. For Gln deprivation experiments, DMEM (11960-044 - Gibco) Gln depleted media was supplemented with Gln (Gemini) at indicated concentration. For glucose deprivation experiments, DMEM (11966025 - Gibco) glucose depleted media was supplemented or not with glucose (Sigma) or treated with 2-Deoxy-D-Glucose (Sigma), at indicated concentration. Cell-permeable dimethyl-α-ketoglutarate (DMα-KG), N-acetyl-L-cysteine (NAC) and Glutathione ethyl ester (GSH) (Cayman) were diluted in PBS immediately before used. Doxycycline (Sigma) was diluted in water, sterilized with a 0.22 μm filter, and stored at – 20 °C. 6-Dizao-5-oxo-L-norleucine (DON) (Cayman) were diluted in PBS. Chloroquine, staurosporine (Sigma), AGI-5198 (ApexBio), and everolimus (LC Laboratories) were diluted in Dimethyl sulfoxide (DMSO) (Sigma).

*Metabolites quantification*

Cells treated with JHU-083 (1 x 10^4^ cells) were used to quantify total ATP, GSH/GSSH, glutamine/Glu and lactate. Metabolites quantification were performed with CellTiter-Glo (Promega), Glutamine/Glutamate-Glo assay (Promega), Lactate-Glo assay (Promega), and GSH/GSSH-Glo assay (Promega) according with manufacture’s protocol. Luminescence was read in Victor-3 automated plate reader (Perkin-Elmer).

*Western blot*

Protein lysates were extracted using RIPA buffer (Sigma) containing protease and phosphatase inhibitor cocktails (ThermoFisher). Protein concentration and Nuclear and cytoplasmic subcellular fractionation were measured using the BCA protein kit assay and NE-PER Nuclear and Cytoplasmic Extraction Reagent, respectively (ThermoFisher). Western blot was performed as previously described ^6^. The following primary antibodies were used: cleaved Poly(ADP-ribose) polymerase (PARP) Asp214, PARP, P21, P27, phospho-S6 ribosomal protein (S6) (ser235/236), S6, phospho-Eukaryotic translation initiation factor 4E (eIF4E)-binding protein (4E-BP1) (thr37/46), phosphor-AKT(ser473), AKT (Cell Signaling Technologies), Cyclin D1, GAPDH, human GLS (Abcam - ab156876), IDH1R132H (Dianova) ACTIN (Sigma), and α-TUBULIN (Santa Cruz Technologies).

*Immunohistochemistry*

Immunohistochemistry (IHC) was performed on formalin-fixed paraffin-embedded sections from xenograft tumors. Immunodetection of IDH1R132H (Dianova), and phospho-S6(ser235/236) (Cell Signaling) were performed on 5 μm sections. Tissue sections were sequentially deparaffinized, rehydrated, and antigen unmasked by boiling the sections in antigen retrieval citrate solution (Biogenex). Sections were blocked and incubated with primary antibody overnight, followed by incubation with biotinylated secondary antibody and streptavidin-conjugated to horseradish peroxidase (Super Sensitive Detection System, Biogenex). Sections were stained with 3’3-diaminobenzidine (DAB) chromogen and counterstained with Gill’s hematoxylin.

*Glucose uptake assay*

Glucose uptake was quantified using the glucose fluorescent analogy 2-(N-(7-Nitrobenz-2-oxa-1,3-diazol-4-yl)Amino)-2-Deoxyglucose (2-NBDG) (Thermo Fisher Scientific). Cells treated with JHU-083 were washed with PBS, and 1 x 10^5^ cells were incubated with 2-NBDG (20 µM) in HEPES-Na buffer for 1 hour, and washed twice with PBS. Fluorescence was read in Victor-3 automated plate reader (Perkin-Elmer), with a 465-nm excitation/ 540-nm emission filter.

**SUPPLEMENTARY FIGURE LEGENDS**

**Supplementary Figure 1. Glucose does not rescue growth inhibition induced by Gln deprivation.** (A) Cell viability was performed in cells cultivated with indicated Gln concentration (72 hours). (B) Cell viability was analyzed in U251 cells cultivated in glutamine-free media supplemented with Glu (72 hours). (C) Colony forming assay was performed in U251 cells cultivated in glutamine-free media supplemented with Glu. (D) U251 cell line was maintained for 24 hours in Gln-deprived media and equal number of cells were analyzed for glucose analog uptake (2-NBDG). (E) Cell viability was performed in U251 cells cultivated in glucose-free media. (F) Cell viability was performed in U251 cells cultivated with Gln in the presence of 2-DG (2 mM) or high glucose concentration (50 mM) for 72 hours. 2-DG: 2-Deoxy-D-Glucose. *P<0.05; **P<0.01; ***P<0.001; ****P<0.0001 vs Control.

**Supplementary Figure 2. Glutaminolysis enzymes and stem-like cell markers in glioma cells.** (A) GLS protein expression was analyzed by Western blot. (B) Schematic representation of *GLS* gene isoforms and strategy to specifically amplify *KGA* (P3 and P4) and *GAC* (P1 and P2) *GLS* isoforms. P2 was designed to cover the exon 14 and exon 15 junction and P4 was designed to cover the exon 14 and exon 16 junction. (C) Representative agarose gel showing the gene expression of *GLS* isoforms, *GLS2*, *NESTIN* (*NES*), and *SRY-box transcription factor 2* (*SOX2*) in glioma cells cultured in serum-free media. JHH273 patient-derived glioma were used as additional control ^2^. NTC: non-template control.

**Supplementary Figure 3. NEAA do not impact in JHU-083 mediated cell growth reduction.** (A) Cell viability was performed in BT142 cells cultivated with indicated NEAA concentration or (B) individually treated with NEAA (1 mM) for 96 hours. NEAA: Non-essential amino acids. (C) Cell viability were performed in U251 in Gln-deprived media and treated in combination with NAC, GSH, Glu, and DM-αKG at indicated concentration for 96 hours.

**Supplementary Figure 4. Targeting Gln metabolism regulates mTOR signaling.** (A) BT142 cells were treated with JHU-083 (10 μM) in combination with Glu (2 mM) for 24 hours and protein expression was analyzed by Western blot. (B) Indicated cell lines were treated with JAM326 for 24 hours and protein expression was analyzed by Western blot. (C) BT142 cells were treated with JHU-083 (10 μM) for 72 hours and protein expression was analyzed by Western blot. HEK293T treated with staurosporine (500 nM) for 24 hours was used as positive control for cleaved PARP detection.

**Supplementary Figure 5.** **Combination of nucleotides or DM-αKG does not rescue growth inhibition induced by JHU-083.** Cell viability was performed in BT142 cells cultivated with indicated metabolites in combination with JHU-083.

**Supplementary Figure 6. Mouse body mass harboring intracranial BT142 glioma.** Body mass was determined weekly in control, JHU-083 (1.9 mg/kg), and JHU-083 (25 mg/kg) groups.

**Supplementary Figure 7. Electropherogram from DNA sequence analysis obtained from SVG cells.**

**REFERENCES**

**1.** Binder ZA, Wilson KM, Salmasi V, et al. Establishment and Biological Characterization of a Panel of Glioblastoma Multiforme (GBM) and GBM Variant Oncosphere Cell Lines. *PLoS One.* 2016; 11(3):e0150271.

**2.** Borodovsky A, Meeker AK, Kirkness EF, et al. A model of a patient-derived IDH1 mutant anaplastic astrocytoma with alternative lengthening of telomeres. *J Neurooncol.* 2015; 121(3):479-487.

**3.** Seltzer MJ, Bennett BD, Joshi AD, et al. Inhibition of glutaminase preferentially slows growth of glioma cells with mutant IDH1. *Cancer Res.* 2010; 70(22):8981-8987.

**4.** Wei S, Wang J, Oyinlade O, et al. Heterozygous IDH1(R132H/WT) created by "single base editing" inhibits human astroglial cell growth by downregulating YAP. *Oncogene.* 2018; 37(38):5160-5174.

**5.** Borodovsky A, Salmasi V, Turcan S, et al. 5-azacytidine reduces methylation, promotes differentiation and induces tumor regression in a patient-derived IDH1 mutant glioma xenograft. *Oncotarget.* 2013; 4(10):1737-1747.

**6.** Yamashita AS, da Costa Rosa M, Borodovsky A, Festuccia WT, Chan T, Riggins GJ. Demethylation and epigenetic modification with 5-azacytidine reduces IDH1 mutant glioma growth in combination with temozolomide. *Neuro Oncol.* 2019; 21(2):189-200.
